# Preeclampsia and Long-Term Risk of Venous Thromboembolism

**DOI:** 10.1001/jamanetworkopen.2023.43804

**Published:** 2023-11-17

**Authors:** Eva Havers-Borgersen, Jawad H. Butt, Marianne Johansen, Olav Bjørn Petersen, Charlotte Kvist Ekelund, Line Rode, Jonas Bjerring Olesen, Lars Køber, Emil L. Fosbøl

**Affiliations:** 1 Department of Cardiology, Rigshospitalet, Copenhagen University Hospital, Copenhagen, Denmark; 2 Department of Obstetrics, Rigshospitalet, Copenhagen University Hospital, Copenhagen, Denmark; 3 Department of Clinical Medicine, University of Copenhagen, Copenhagen, Denmark; 4 Department of Cardiology, Herlev and Gentofte Hospital, Gentofte, Denmark

## Abstract

**Importance:**

As venous thromboembolism (VTE) remains one of the leading causes of maternal mortality, identifying women at increased risk of VTE is of great importance. Preeclampsia is a pregnancy-induced hypertensive disorder with generalized endothelial dysfunction. Some studies suggest that preeclampsia is associated with an increased risk of VTE, but much controversy exists.

**Objective:**

To examine the association between preeclampsia and the risk of VTE during pregnancy, during the puerperium, and after the puerperium.

**Design, Setting, and Participants:**

This observational cohort study used Danish nationwide registries to identify all eligible primiparous women who gave birth in Denmark from January 1, 1997, to December 31, 2016. The women were followed up from primiparous pregnancy to incident VTE, emigration, death, or the end of the study (December 31, 2016). Statistical analyses were carried out from January to May 2023.

**Exposure:**

Preeclampsia during primiparous pregnancy.

**Main Outcomes and Measure:**

The main outcome was incident VTE, and the secondary outcome was all-cause mortality.

**Results:**

A total of 522 545 primiparous women (median age, 28 years [IQR, 25-31 years]) were included, and 23 330 (4.5%) received a diagnosis of preeclampsia. Women with preeclampsia were of similar age to women without preeclampsia but had a higher burden of comorbidities. During a median follow-up of 10.2 years (IQR, 5.2-15.4 years), preeclampsia was associated with a higher incidence of VTE compared with no preeclampsia (incidence rate, 448.8 [95% CI, 399.9-503.5] vs 309.6 [95% CI, 300.6-319.9] per 1000 patient-years, corresponding to an unadjusted hazard ratio [HR] of 1.45 [95% CI, 1.29-1.63] and an adjusted HR of 1.43 [95% CI, 1.27-1.61]). When stratified according to the subcategories of VTE, preeclampsia was associated with an increased rate of deep vein thrombosis (unadjusted HR, 1.51 [95% CI, 1.32-1.72] and adjusted HR, 1.49 [95% CI, 1.31-1.70]) as well as pulmonary embolism (unadjusted HR, 1.39 [95% CI, 1.09-1.76]; adjusted HR, 1.36 [95% CI, 1.08-1.73]). These findings held true in landmark analyses during pregnancy, during the puerperium, and after the puerperium.

**Conclusions and Relevance:**

This cohort study suggests that preeclampsia was associated with a significantly increased risk of VTE during pregnancy, during the puerperium, and after the puerperium, even after thorough adjustment. Future studies should address how to improve the clinical management of women with a history of preeclampsia to prevent VTE.

## Key Points

**Question:**

Are women with a history of preeclampsia at increased risk of venous thromboembolism?

**Findings:**

This nationwide cohort study of 522 545 primiparous women found that preeclampsia was associated with an increased risk of venous thromboembolism during pregnancy, during the puerperium, and after the puerperium, even after comprehensive adjustment for potential confounders.

**Meaning:**

As venous thromboembolism remains one of the leading causes of maternal death, these findings suggest that increased awareness is important for women with a history of preeclampsia.

## Teaser

This cohort study examines the association between preeclampsia and the risk of venous thromboembolism during pregnancy, during the puerperium, and after the puerperium.

## Introduction

Preeclampsia affects 4% to 5% of pregnancies worldwide and is defined by new-onset hypertension accompanied by multiorgan involvement, including uteroplacental dysfunction.^1^ The exact cause and pathogenesis of preeclampsia remain unknown, but there is consensus that generalized endothelial dysfunction plays an important role.^1^ Thrombophilia can be either acquired or inherited and has been reported to be associated with an increased risk of preeclampsia.^1,2,3^ Likewise, thrombophilia is associated with an increased risk of venous thromboembolism (VTE), including deep vein thrombosis and pulmonary embolism—an association also observed among women during pregnancy and puerperium.^4,5^ Besides thrombophilia, known VTE risk factors include obesity, heart failure, cancer, inflammatory and autoimmune diseases, and venous insufficiency.^2,3^ As VTE remains one of the leading causes of maternal mortality,^4^ identifying women at increased risk is of great importance in terms of prevention.

The association between preeclampsia and the risk of VTE during pregnancy and in the puerperium has been thoroughly examined, yet studies are conflicting. Some studies report an association between preeclampsia and an increased risk of VTE during pregnancy and in the puerperium,^5,6^ while others report an association only in the puerperium but not during pregnancy^7,8^ or no association at all.^9,10^ Thus, much controversy still exists. Only one of these studies adjusted for relevant risk factors including thrombophilia,^5^ whereas others did not adjust at all or only for demographic data.^6,7,8,9,10^ Data on the association between preeclampsia and the long-term risk of VTE are scarce. To date, 4 studies have examined the long-term association.^6,11,12,13^ They all reported an increased long-term risk of VTE among women with preeclampsia but none of these 4 studies adjusted for VTE risk factors. Thus, current data suggest that a history of preeclampsia is associated with an increased long-term risk of VTE, but whether preeclampsia is part of a causal chain or is an independent risk factor for VTE remains unclear. By adjusting for VTE risk factors, it is possible to minimize residual confounding and thus potentially approach causality. This nationwide cohort study investigated the association between preeclampsia and the risk of VTE both in association with pregnancy and the puerperium and in the long term, accounting for established VTE risk factors.

## Methods

### Data Sources

All Danish residents are assigned a unique and permanent civil registration number, allowing accurate linkage of nationwide administrative registries at an individual level. For this study, data from 5 Danish administrative registries were obtained. The Danish National Patient Registry holds information on all hospital admissions and outpatient contacts according to the *International Classification of Diseases, Eighth Revision* and the *International Statistical Classification of Diseases and Related Health Problems, Tenth Revision* (*ICD-10*) and surgical procedures according to the NOMESCO (Nordic Medico-Statistical Committee) Classification of Surgical Procedures.^14^ The Danish Civil Registration System holds data on birth date, sex, ethnicity, and whether a person is alive and a resident in Denmark, disappeared (persons whose residence is unknown to Danish authorities), emigrated, or dead, along with the date of these events.^15^ Immigration status is defined by Statistics Denmark and based on the place of birth and citizenships of the individuals themselves and their parents. The Danish Medical Birth Register contains information on all births in Denmark, including maternal demographic data, pregnancy-related variables, variables related to birth, and information on the newborn.^16^ The Danish National Prescription Registry contains information on dispensing date, strength, and quantity of all claimed drug prescriptions in Denmark.^17^ The Danish Register of Causes of Death holds information on the date, place, and manner of death, as well as the underlying cause coded according to the *ICD-10*, based on death certificates.^18^ All the registries are validated and of high quality, as described in detail previously.^15,17,19,20,21^ In Denmark, registry-based studies that are conducted for the sole purpose of statistics and scientific research do not require ethical approval or informed consent by law. However, this study was approved by the Capital Region of Denmark (approval No. P-2019-399) in accordance with the General Data Protection Regulation. Due to rules on anonymity by Statistics Denmark, exact numbers on data from the registries are known but cannot be reported if the number of patients is fewer than 4. This study followed the Strengthening the Reporting of Observational Studies in Epidemiology (STROBE) reporting guideline.

### Study Population

All Danish women giving birth for the first time (ie, primiparous) during the period from January 1, 1997, to December 31, 2016, were identified. Women with a history of VTE were excluded. Preeclampsia was defined as a diagnosis of preeclampsia, eclampsia, or the HELLP (hemodialysis, elevated liver enzymes, and low platelets) syndrome using the following *ICD-10* codes: DO11, DO14-DO15, BKHE1, and BKHE2. Preeclampsia was further stratified into early onset and late onset (ie, birth <34 vs ≥34 weeks’ gestation, respectively) as we used gestational age at time of birth as a proxy for severity of preeclampsia.

### Covariates

Comorbidities were defined as inpatient and outpatient diagnoses any time prior to pregnancy. Concomitant pharmacotherapy was defined as a filled prescription within 1 year prior to pregnancy. Patients with diabetes and hypertension were identified using claimed drug prescriptions and/or diagnosis codes as described previously.^22^ Risk factors for VTE were defined based on the European guidelines and included obesity, thrombophilia (including antiphospholipid syndrome), heart failure, cancer, inflammatory and autoimmune diseases, and venous insufficiency.^2,3^ The eTable in Supplement 1 depicts *ICD-10* codes and Anatomical Therapeutic Chemical codes.

### Outcomes

The primary outcome was incident VTE. Venous thromboembolism was defined as a composite of pulmonary embolism and deep vein thrombosis using the following *ICD-10* codes for pulmonary embolism (DI26, DO882C, DO882D, and DO882E) and deep vein thrombosis (DI801-803, DI808, DI809, DI821-823, DI828, DI829, DO222, DO223, DO225, DO870, DO871, and DO873). Most often, only the most severe diagnosis is registered. Thus, patients with pulmonary embolism are registered with a diagnosis as such regardless of whether there is coexisting deep vein thrombosis, but only patients with deep vein thrombosis without concomitant pulmonary embolism are given a diagnosis of deep vein thrombosis. The diagnosis of VTE has previously been validated with high positive predictive values ranging from 86% to 90%.^23,24,25^ The secondary outcome was all-cause mortality.

### Statistical Analysis

Statistical analyses were carried out from January to May 2023. The women were followed up from the start of their primiparous pregnancy until the outcome of interest, emigration, or the end of the study period (December 31, 2016). Women without preeclampsia in the primiparous pregnancy who developed preeclampsia in a later pregnancy were censored on the day of preeclampsia diagnosis; hence, follow-up was stopped. Women who developed recurrent preeclampsia were not censored.

In supplementary analyses, 3 landmark analyses were performed examining the risks of VTE in 3 time spans: (1) during pregnancy, (2) during the puerperium (ie, 6 weeks after birth), and (3) after the puerperium. When examining the risk of VTE during and after the puerperium, patients were excluded if they developed VTE, died, or emigrated before end of pregnancy or before the end of puerperium. In an additional analysis, the risk of VTE was compared among women with early-onset vs late-onset preeclampsia (birth <34 vs ≥34 weeks’ gestation, respectively).

Baseline characteristics of the women were reported by use of frequencies and percentages for categorical variables and median values with IQRs for continuous variables. Crude incidence rates (IRs) of VTE and all-cause mortality among women with vs without preeclampsia were calculated as the number of events per 1000 person-years. Cumulative incidences of VTE were estimated using the Aalen-Johansen estimator incorporating the competing risks of death and preeclampsia in a subsequent pregnancy. Differences between groups were assessed using the Gray test.^19^ Cumulative incidences of all-cause mortality were estimated using the Kaplan-Meier estimator. Differences between groups were assessed using the log-rank test. Furthermore, in cause-specific Cox proportional hazards regression models, the unadjusted and adjusted rates of VTE and all-cause mortality were calculated as hazard ratios (HRs) with 95% CIs. The adjusted HRs were calculated by including known VTE risk factors (ie, older age [>40 years], obesity, thrombophilia including antiphospholipid syndrome, heart failure, cancer, inflammatory and autoimmune diseases, and venous insufficiency) in a multivariable Cox proportional hazards regression model. Cause-specific Cox proportional hazards regression models were used to alleviate the bias created by censoring.^26^ Due to missing data on body mass index (BMI; calculated as weight in kilograms divided by height in meters squared) (195 839 of 522 545 [37.5%]), obesity (BMI ≥25) was not included in the main adjusted analysis. However, a sensitivity analysis excluding patients with missing data on BMI and including obesity in the adjusted analyses was performed.

All statistical analyses were performed with SAS statistical software, version 9.4 (SAS Institute Inc). A 2-sided *P* < .05 was considered statistically significant.

To test the robustness of our findings, we performed a sensitivity analysis excluding all patients with missing data on BMI to adjust for obesity (BMI ≥25) in the multivariable analyses. We chose to dichotomize the BMI to align with the European guidelines, in which obesity is defined as a VTE risk factor.^2,3^ Furthermore, we performed a second sensitivity analysis, examining the incidence of VTE in a cause-specific multivariable Cox proportional hazards regression model including preeclampsia as a time-dependent variable, thus including those who developed preeclampsia in a subsequent pregnancy.

## Results

### Baseline Characteristics

**Figure 1.**
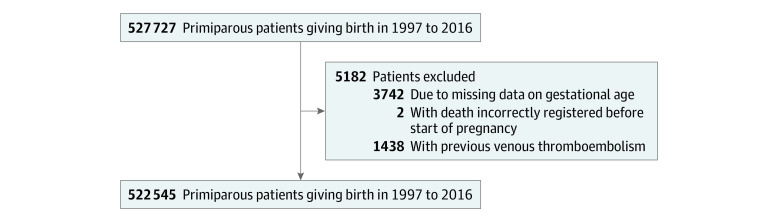
Selection of the Study Population

In total, 527 727 primiparous women were eligible; after exclusion due to previous VTE or missing data, 522 545 women with a median age of 28 years (IQR, 25 to 31 years) were included in the study (Figure 1). Of these women, 23 330 (4.5%) received a diagnosis of preeclampsia during their primiparous pregnancy; 2083 of these women (8.9%) had early-onset preeclampsia (ie, preeclampsia with birth <34 weeks’ gestation). The baseline characteristics of the women with or without preeclampsia are provided in the Table. Overall, women with preeclampsia had a higher burden of comorbidities, including diabetes (785 of 23 330 [3.4%] vs 7288 of 499 215 [1.5%]) and inflammatory and autoimmune diseases (867 of 23 330 [3.7%] vs 9910 of 499 215 [2.0%]).

**Table.** Baseline Characteristics Among Women With or Without Preeclampsia Including Pregnancy and Birth Data as Well as Comorbidities and Use of Pharmacotherapy Prior to Primiparous Pregnancy

Characteristic	Women, No. (%)	
	Preeclampsia (n = 23 330)	No preeclampsia (n = 499 215)
Maternal age, y		
<25	4586 (19.7)	108 631 (21.8)
25-29	9452 (40.5)	209 920 (42.1)
30-34	5554 (27.5)	117 331 (26.7)
≥35	2874 (12.3)	47 216 (9.5)
Median (IQR), y	28 (25-32)	28 (25-31)
Smoking status		
Nonsmoker	18 557 (79.5)	384 274 (77.0)
Smoker	1862 (8.0)	58 092 (11.6)
Quit smoking during pregnancy	825 (3.5)	16 699 (3.3)
Unknown	2086 (8.9)	40 150 (8.0)
BMI		
<18.5	404 (1.7)	15 390 (3.1)
18.5-24	7818 (33.5)	205 178 (41.1)
25-29	3814 (16.3)	59 513 (11.9)
30-34	1836 (7.9)	21 096 (4.2)
>34	1300 (5.6)	10 357 (2.1)
Unknown	8158 (35.0)	187 681 (37.6)
Immigration status		
Native Danish	21 091 (90.4)	429 058 (85.9)
Immigrant	1909 (8.2)	61 283 (12.3)
Descendant from immigrant	327 (1.4)	8747 (1.8)
Comorbidities prior to pregnancy		
Diabetes	785 (3.4)	7288 (1.5)
Pregestational hypertension	471 (2.0)	1980 (0.4)
Alcohol abuse	227 (1.0)	4821 (1.0)
Thrombophilia	14 (0.1)	298 (0.1)
Venous insufficiency	4 (0.02)	17 (0.003)
Inflammatory and autoimmune diseases	867 (3.7)	9910 (2.0)
Heart failure	11 (0.1)	126 (0.03)
Cancer	114 (0.5)	2171 (0.4)
Chronic kidney disease	108 (0.5)	571 (0.1)
Pharmacotherapy within 1 y prior to pregnancy		
Oral contraception pill	9619 (41.2)	201 364 (40.3)
Low-dose aspirin	57 (0.2)	451 (0.1)
Statins	97 (0.4)	608 (0.1)
NSAID	3905 (16.7)	71 812 (14.4)
Primiparous birth		
Gestational age, median (IQR), wk	38.9 (37.1-40.1)	40.1 (39.0-41.0)
Preterm birth (<37 wk gestation)	5351 (22.9)	33 993 (6.8)
Mode of delivery		
Vaginal delivery	7877 (33.8)	309 794 (62.1)
Instrumental vaginal delivery	2907 (12.5)	63 849 (12.8)
Cesarean delivery	9041 (38.8)	93 968 (18.8)
Unknown	1903 (8.2)	20 030 (4.0)
Multiple births	1602 (6.9)	11 574 (2.3)
Birth weight, median (IQR), g	3165 (2600-3610)	3448 (3100-3775)
Stillbirth	101 (0.4)	1839 (0.4)
5-min Apgar score <7	400 (1.7)	4765 (1.0)
Neonatal death	153 (0.7)	1850 (0.4)
Neonatal length of hospital admission, median (IQR), d	5 (3-8)	3 (2-5)
NICU admission	6364 (27.3)	51 897 (10.4)
Length of stay, median (IQR), d	11 (3-24)	5 (1-14)
Maternal death	10 (0.04)	35 (0.01)
Maternal length of hospital admission, median (IQR), d	6 (4-10)	4 (3-5)

Abbreviations: BMI, body mass index (calculated as weight in kilograms divided by height in meters squared); NICU, neonatal intensive care unit; NSAID, nonsteroidal anti-inflammatory drug.

### Venous Thromboembolism

**Figure 2.**
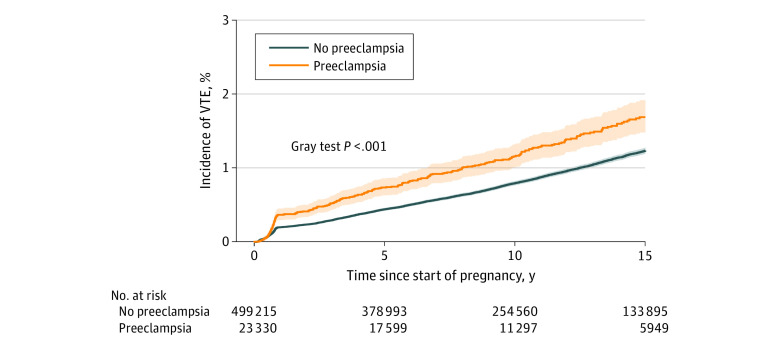
Long-Term Cumulative Incidences of Venous Thromboembolism (VTE) Among Women With vs Without Preeclampsia in Their Primiparous Pregnancy Shaded areas indicate 95% CIs.

During a median follow-up period of 10.2 years (IQR, 5.2-15.4 years), 4668 women (0.9%) developed VTE. The median time from primiparous pregnancy to incident VTE was 5.8 years (IQR, 1.6-10.9 years). During follow-up, preeclampsia was associated with a significantly higher incidence of overall VTE compared with no preeclampsia (IR, 448.8 per 1000 patient-years [95% CI, 399.9-503.5 per 1000 patient-years] vs 309.6 per 1000 patient-years [95% CI, 300.6-319.9 per 1000 patient-years]; Figure 2). Pulmonary embolism accounted for 1228 of 4668 cases of VTE (26.3%), whereas deep vein thrombosis accounted for 3682 of 4668 cases of VTE (78.9%); thus, 242 of 4668 women (5.2%) received a diagnosis of both conditions.

**Figure 3.**
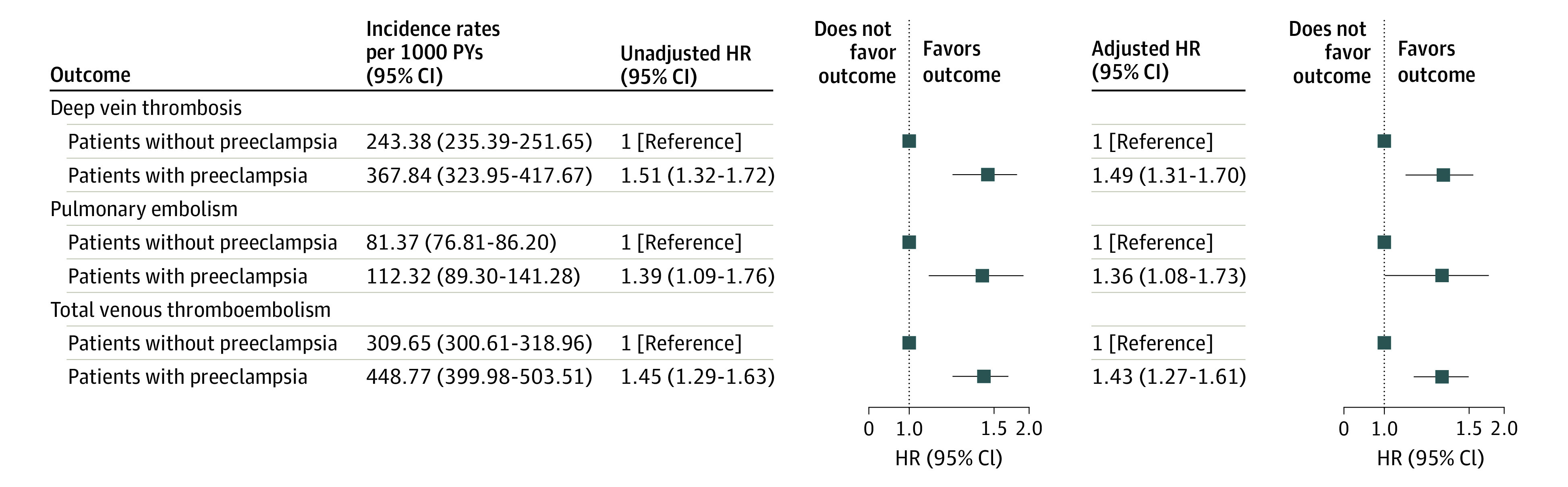
Rates of Deep Vein Thrombosis, Pulmonary Embolism, and Overall Venous Thromboembolism Among Women With vs Without Preeclampsia in Their Primiparous Pregnancy Adjusted for thrombophilia including antiphospholipid syndrome, heart failure, cancer, inflammatory and autoimmune diseases, and venous insufficiency. HR indicates hazard ratio; and PY, patient-years.

Figure 3 shows the association of preeclampsia with deep vein thrombosis, pulmonary embolism, and VTE overall. Women with preeclampsia had a significantly higher rate of deep vein thrombosis (unadjusted HR, 1.51 [95% CI, 1.32-1.72]; adjusted HR, 1.49 [95% CI, 1.31-1.70]), pulmonary embolism (unadjusted HR, 1.39 [95% CI, 1.09-1.76]; adjusted HR, 1.36 [95% CI, 1.08-1.73]), and overall VTE (unadjusted HR, 1.45 [95% CI, 1.29-1.63]; adjusted HR, 1.43 [95% CI, 1.27-1.61]) than did women without preeclampsia. The incidence of VTE overall was associated mainly with the numerically greater incidence of deep vein thrombosis. Among women with preeclampsia, the factors associated with VTE included thrombophilia (HR 9.13 [95% CI, 1.28-65.35]).

### Supplementary Analyses

Women with preeclampsia had a significantly higher incidence of VTE compared with those without preeclampsia in all 3 landmark analyses. eFigure 1A, 1B, and 1C in Supplement 1 show the cumulative incidences of VTE during the 3 time spans: (1) during pregnancy, (2) in the puerperium, and (3) after the puerperium, respectively. eFigure 2 in Supplement 1 shows the association of preeclampsia with deep vein thrombosis, pulmonary embolism, and VTE overall stratified in the 3 time spans. In unadjusted analysis as well as adjusted analysis, preeclampsia was associated with a higher rate of deep vein thrombosis, pulmonary embolism, and overall VTE. Only pulmonary embolism after the puerperium showed no statistical significance, but a trend toward a difference was seen. Among women with preeclampsia, the incidence of VTE was substantially higher among those with early-onset vs late-onset preeclampsia (51 of 2083 [2.5%] vs 239 of 21 247 [1.1%]) (eFigure 3 in Supplement 1).

### Mortality

During follow-up, 1956 women (0.4%) died, and the incidence of mortality was significantly higher among women with vs without preeclampsia (IR, 175.1 [95% CI, 145.7-210.4] vs 128.7 [95% CI, 123.0-134.7]) (eFigure 4 in Supplement 1), corresponding to an unadjusted HR of 1.38 (95% CI, 1.14-1.67) and an adjusted HR of 1.35 (95% CI, 1.12-1.63).

Women with preeclampsia who developed VTE had a similar risk of mortality compared with those without preeclampsia who developed VTE (9 of 290 [3.1%] vs 148 of 4378 [3.4%]; unadjusted HR, 1.01 [95% CI, 0.52-1.99]; adjusted HR, 0.92 [95% CI, 0.47-1.81]). On the other hand, women with preeclampsia who developed VTE had a significantly higher risk of mortality compared with those with preeclampsia who did not develop VTE (2 of 290 [3.1%] vs 105 of 23 040 [0.5%]; unadjusted HR, 5.46 [95% CI, 2.76-10.78]; adjusted HR, 5.54 [95% CI, 2.74-10.72]).

### Sensitivity Analyses

In the first sensitivity analysis, 195 839 patients were excluded due to missing data on BMI, with similar findings; the incidence of VTE was significantly higher among women with vs without preeclampsia (149 of 15 172 [1.0%] vs 2044 of 311 534 [0.7%], corresponding to an adjusted HR of 1.38 [95% CI, 1.17-1.64]). Likewise, in the second sensitivity analysis examining the incidence of VTE in a cause-specific multivariable Cox proportional hazards regression model including preeclampsia as a time-dependent variable, similar findings were obtained, as the incidences of VTE were 1.3% (368 of 28 448) vs 0.9% (4300 of 494 097) among women with vs without preeclampsia (adjusted HR, 1.26 [95% CI, 1.12-1.43]).

## Discussion

In this large nationwide cohort study, we assessed the risk of VTE among women with vs without preeclampsia during their primiparous pregnancy. The study revealed the following major finding: women with vs without preeclampsia in their primiparous pregnancy, and in particular those with early-onset preeclampsia, had a significantly higher long-term risk of VTE, including deep vein thrombosis as well as pulmonary embolism. This finding was true even after comprehensive adjustment for known VTE risk factors. This finding also held true in landmark analyses during pregnancy and during and after the puerperium.

Venous thromboembolism remains one of the leading causes of maternal mortality^4^; therefore, identifying women at increased risk of VTE is of great importance. The pathogenesis of preeclampsia is complex and includes placental dysfunction, causing stressed syncytiotrophoblast. The stressed syncytiotrophoblast then releases factors that cause maternal systemic inflammation, endothelial dysfunction, reduced vasodilation, and thrombosis.^1^ Thus, one could hypothesize that preeclampsia itself may be associated with the risk of VTE. Moreover, thrombophilia has been reported to be associated with an increased risk of preeclampsia^27,28,29^ as well as VTE^30,31^; hence, the 2 conditions may share some pathogenetic characteristics. It is essential to investigate whether an association between preeclampsia and VTE is causal or not (ie, due to common underlying risk factors including thrombophilia) or whether preeclampsia is an independent risk factor. However, this remains challenging in observational studies, but by comprehensive adjustment for potential confounders, it is a fairer presentation of the association, although the risk of residual confounding cannot be completely omitted. Previous studies on the association between preeclampsia and risk of VTE during pregnancy and in the puerperium are conflicting,^5,6,7,8,9,10^ and only one of these previous studies adjusted for selected risk factors including thrombophilia.^5^ The latter Danish study by Virkus et al^5^ found an association between preeclampsia and VTE during pregnancy and during the puerperal period. In addition, 4 studies have examined the association between preeclampsia and the long-term risk of VTE, but none of these have taken known VTE risk factors into account.^6,11,12,13^ In 1997, Hannaford et al^11^ found an increased long-term risk of VTE after adjustment for age, smoking, and social class among women (N = 17 202) with a history of preeclampsia during a mean follow-up period of 12.5 years. However, the study had several limitations, including the fact that the diagnosis of VTE was made by general practitioners with no specifications of diagnostic criteria. In 2003, van Walraven et al^12^ (N = 297 037) and Kestenbaum et al^13^ (N = 113 454) also found an association between preeclampsia and long-term risk of VTE in unadjusted analyses over mean follow-up periods of 3 and 7.8 years, respectively. Most recently, in 2020, Scheres et al^6^ investigated the long-term risk of VTE over a mean follow-up period of 13.7 years and found an increased risk of VTE after adjustment for number of previous pregnancies, age, and self-reported descent. To our knowledge, this present study is the first to show an association between preeclampsia, and in particular early-onset preeclampsia, and long-term risk of VTE after thorough adjustment for known VTE risk factors. We found that the association held true during pregnancy, during puerperium, and after the puerperium. This finding indicates that preeclampsia is a risk factor for VTE per se. This may be due to long-lasting endothelial dysfunction caused by preeclampsia. In addition, we found that women with a history of preeclampsia and thrombophilia were at further increased risk of VTE; thus, particular attention should be paid to these women. Data from a large systematic review and meta-analysis and a randomized clinical trial have demonstrated a reduction in preeclampsia with the use of low-dose aspirin.^32,33^ By identifying women at increased risk of preeclampsia and prescribing low-dose aspirin, not only may preeclampsia be prevented, but the preeclampsia-associated increased risk of VTE may also be reduced. As women with preeclampsia are also at increased long-term risk of other cardiovascular outcomes including hypertension, hypercholesterolemia, heart failure, coronary heart disease, stroke, dementia, peripheral arterial disease, and type 2 diabetes,^34,35,36,37,38,39,40,41^ this finding stresses the importance of increased attention and further research in prevention and treatment of preeclampsia.

### Strengths and Limitations

This study has some strengths. The main strength is the completeness of data in a large nationwide cohort of all primiparous women in a 20-year observation period. The Danish health care system, funded by taxes, provides equal access to health care services for all residents regardless of socioeconomic or insurance status, which eliminates the risk of selection bias. Use of prospectively recorded register data eliminates the possibility of recall bias.

However, the findings in this study should be viewed in the context of its limitations. The study did not include pregnancy losses occurring before 20 weeks’ gestation because data regarding miscarriages until that stage were unavailable. The accuracy of the data relies on a correct diagnosis and coding in nationwide administrative registries, which has previously been validated.^15,17,19,20,21,23^ The preeclampsia diagnosis has previously been validated with a sensitivity of 69%, a specificity of more than 99%, and a positive predictive value of 74% overall and 100% for severe preeclampsia.^42^ Thus, the potential misclassification is therefore most likely of negligible impact. Thrombophlebitis and deep vein thrombosis are grouped together into the common outcome deep vein thrombosis as these conditions are part of the same spectrum of disease. However, this grouping may result in an overdiagnosis of deep vein thrombosis. Unfortunately, data on ultrasonography findings, blood samples, history of immobilization or long-haul flights, family history of VTE, and use of contraception after birth were not available. The women were followed up during a 20-year period, but some may have developed VTE after the end of the follow-up period. Hence, the rate of VTE may be altered if we had access to more recent data. Women with vs without preeclampsia differed in terms of mode of delivery. More women with preeclampsia delivered by cesarean birth than did women without preeclampsia. This difference may have had an association with the risk of VTE during the early puerperium but presumably not with the long-term risk. Moreover, we did not have access to data on pharmacotherapy, including thromboprophylaxis administered during hospitalization. The observational nature of this study precludes the assessment of cause-effect relationships. The influence of potential confounders, including potentially applied preventive measures and yet-undiagnosed thrombophilia and, thus, residual confounding, cannot be omitted despite adjustment for potential confounders in the Cox proportional hazards regression models. Likewise, the risk of unidentified interactions cannot be omitted.

## Conclusion

In this cohort study, we found an association between preeclampsia and long-term VTE risk, which held true after comprehensive adjustment for known VTE risk factors. This association also persisted in landmark analyses during pregnancy, during the puerperium, and in the time after the puerperium. As women with preeclampsia also have been identified as being at increased long-term risk of other cardiovascular outcomes, this finding stresses the importance of increased attention and further research in prevention and treatment of preeclampsia.

## Notes

Supplement 1. **eTable.** ICD-10, ATC-, and SKS-Codes **eFigure 1A.** Cumulative Incidences of Venous Thromboembolism During Pregnancy Among Women With vs Without Preeclampsia **eFigure 1B.** Cumulative Incidences of Venous Thromboembolism During the Puerperium (ie, 6 Weeks Following Birth) Among Women With vs Without Preeclampsia During Their Primiparous Pregnancy **eFigure 1C.** Cumulative Incidences of Venous Thromboembolism Following the Puerperium (ie, 6 Weeks After Birth) Among Women With vs Without Preeclampsia During Their Primiparous Pregnancy **eFigure 2.** Rates of Deep Vein Thrombosis, Pulmonary, and Overall Venous Thromboembolism Among Women With and Without Preeclampsia During Pregnancy, During the Puerperium, and Following the Puerperium **eFigure 3.** Cumulative Incidences of Venous Thromboembolism Among Women Without Preeclampsia, With Early-Onset (<34 Weeks’ Gestation) Preeclampsia and Late-Onset (>34 Weeks’ Gestation) Preeclampsia **eFigure 4.** Cumulative Incidences of Mortality Among Women With and Without Preeclampsia

Supplement 2.
Data Sharing Statement
