# Miscalculation of Incidence Rates

**DOI:** 10.1001/jamanetworkopen.2023.54306

**Published:** 2024-01-11

In the Original Investigation titled “Preeclampsia and Long-Term Risk of Venous Thromboembolism,”^1^ published November 17, 2023, a software macro error resulted in a miscalculation of incidence rates for pulmonary embolism, deep vein thrombosis, and total venous thromboembolism that affected findings reported in the Abstract, main Results section, and Figure 3. This article has been corrected.^1^
