# Supplementary material for: Preeclampsia and Long-Term Risk of Venous Thromboembolism
Source: JAMA Netw Open. 2023 Nov 17;6(11):e2343804. doi: 10.1001/jamanetworkopen.2023.43804 (PMC10656639; doi:10.1001/jamanetworkopen.2023.43804)
Supplement: Supplement 1. — eTable. ICD-10, ATC-, and SKS-Codes eFigure 1A. Cumulative Incidences of Venous Thromboembolism During Pregnancy Among Women With vs Without Preeclampsia eFigure 1B. Cumulative Incidences of Venous Thromboembolism During the Puerperium (ie, 6 Weeks Following Birth) Among Women With vs Without Preeclampsia During Their Primiparous Pregnancy eFigure 1C. Cumulative Incidences of Venous Thromboembolism Following the Puerperium (ie, 6 Weeks After Birth) Among Women With vs Without Preeclampsia During Their Primiparous Pregnancy eFigure 2. Rates of Deep Vein Thrombosis, Pulmonary, and Overall Venous Thromboembolism Among Women With and Without Preeclampsia During Pregnancy, During the Puerperium, and Following the Puerperium eFigure 3. Cumulative Incidences of Venous Thromboembolism Among Women Without Preeclampsia, With Early-Onset (<34 Weeks’ Gestation) Preeclampsia and Late-Onset (>34 Weeks’ Gestation) Preeclampsia eFigure 4. Cumulative Incidences of Mortality Among Women With and Without Preeclampsia [file jamanetwopen-e2343804-s001.pdf]

Supplementary Online Content

Havers-Borgersen E, Butt JH, Johansen M, et al. Preeclampsia and long-term risk of venous thromboembolism. *JAMA Netw Open.* 2023;6(11):e2343804. doi:10.1001/jamanetworkopen.2023.43804

eTable. ICD-10, ATC-, and SKS-Codes

eFigure 1A. Cumulative Incidences of Venous Thromboembolism During Pregnancy Among Women With vs Without Preeclampsia

eFigure 1B. Cumulative Incidences of Venous Thromboembolism During the Puerperium (ie, 6 Weeks Following Birth) Among Women With vs Without Preeclampsia During Their Primiparous Pregnancy

eFigure 1C. Cumulative Incidences of Venous Thromboembolism Following the Puerperium (ie, 6 Weeks After Birth) Among Women With vs Without Preeclampsia During Their Primiparous Pregnancy

eFigure 2. Rates of Deep Vein Thrombosis, Pulmonary, and Overall Venous Thromboembolism Among Women With and Without Preeclampsia During Pregnancy, During the Puerperium, and Following the Puerperium

eFigure 3. Cumulative Incidences of Venous Thromboembolism Among Women Without Preeclampsia, With Early-Onset (<34 Weeks' Gestation) Preeclampsia and Late-Onset (≥34 Weeks' Gestation) Preeclampsia

eFigure 4. Cumulative Incidences of Mortality Among Women With and Without Preeclampsia

This supplementary material has been provided by the authors to give readers additional information about their work.

© 2023 Havers-Borgersen E et al. *JAMA Network Open.*

eTable. ICD-10, ATC-, and SKS-Codes

**Comorbidities**	**ICD-10 and ATC codes**
Diabetes mellitus	ICD-10: DE10-14<br>*or*<br>ATC: A10
Pregestational hypertension	ICD-10: DI10-DI15<br>*or* ≥2 of the following drugs (ATC-codes):<br>• Non-loop diuretics: C02L, C03A, C03B, C03D, C03E, C03X, C07B, C07C, C07D, C08G, C02DA, C03EA, C03EB, C09BA, C09DA, C09XA52<br>• Loop diuretics: C03C<br>• Beta-blockers: C07A, C07B, C07C, C07D, C07D<br>• Calcium channel blockers: C08, C09BB, C09DB<br>• Adreno-blockers: C02A, C02B, CO2C<br>• Vasodilators: C02DB, C02DD, C02DG<br>• RAS inhibitors: C09AA, C09BA, C09BB, C09CA, C09DA, C09DB
Alcohol abuse	ICD-10: DK860, DE244, DG312, DI426, D03543, DZ721, DG621, DK292, DG312E
Thrombophilia	DD685, DD686
Venous insufficiency	DI872
Inflammatory and autoimmune diseases	DK50-52, DM352, DE10, DM32, DL93, DM33, DL40, DM02, DI00, DI01, DM05, DM34, DM51, DE271-273, DM459, DD590-591, DM352, DI091-092, DK900, DE050, DD693, DK754, DG359, DG700, DD510, DM300, DM315, DK743, DM091, DM092, DM069, DM068, DM080, DL940, DM350, DM313, DM316, DM315
Heart failure	ICD-10: DI43, DI50, DJ81, DI110, DI130, DI132
Cancer	ICD-10: DC00-DC97
Venous thromboembolism	
Pulmonary embolism	DI26, DO882C, DO882D, DO882E
Deep vein thrombosis	DI801-803, DI808-809, DI821-823, DI828, DI829, DO222, DO223, DO225, DO870, DO871, DO873
**Pharmacotherapy**	**ATC-codes**
Oral contraception pill	G03AA
Calcium channel blockers	C08, C09BB, C09DB
Beta blockers	C07A, C07B, C07C, C07D, C07D
Non-loop diuretics	C02L, C03A, C03B, C03D, C03E, C03X, C07B, C07C, C07D, C08G, C02DA, C03EA, C03EB, C09BA, C09DA, C09XA52
Loop diuretics	C03C
Thyroid medication	H03
Acetylic acid	B01AC06, N02BA01
Statins	C10AA
NSAID	M01A but not M01AX05

© 2023 Havers-Borgersen E et al. *JAMA Network Open.*

eFigure 1A. Cumulative Incidences of Venous Thromboembolism During Pregnancy Among Women With vs Without Preeclampsia

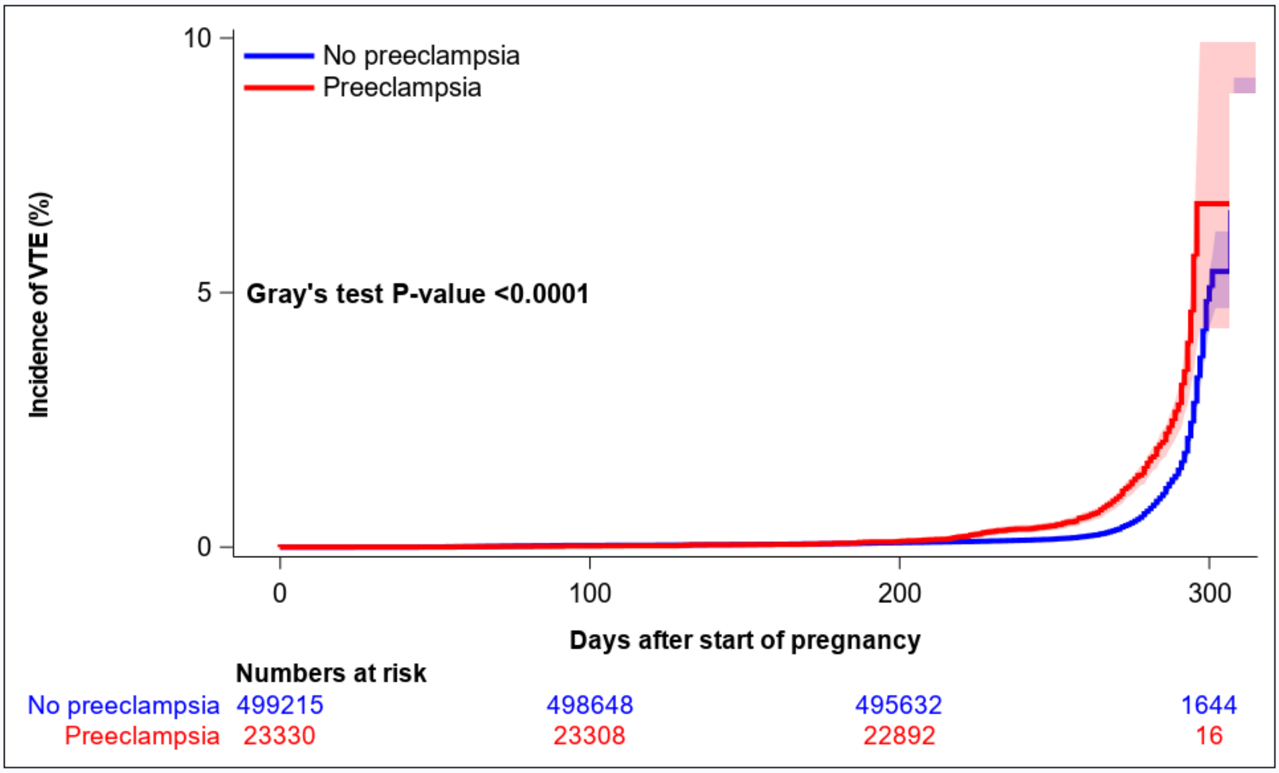

eFigure 1B. Cumulative Incidences of Venous Thromboembolism During the Puerperium (ie, 6 Weeks Following Birth) Among Women With vs Without Preeclampsia During Their Primiparous Pregnancy

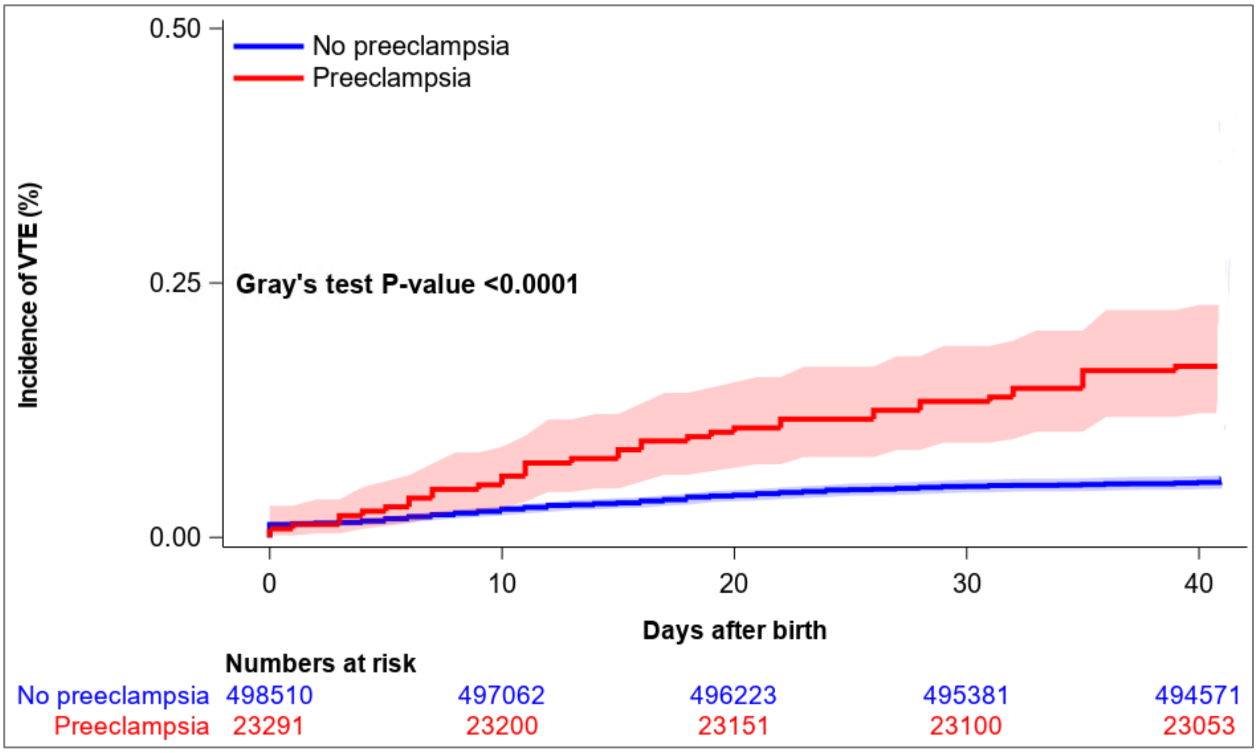

© 2023 Havers-Borgersen E et al. *JAMA Network Open.*

eFigure 1C. Cumulative Incidences of Venous Thromboembolism Following the Puerperium (ie, 6 Weeks After Birth) Among Women With vs Without Preeclampsia During Their Primiparous Pregnancy

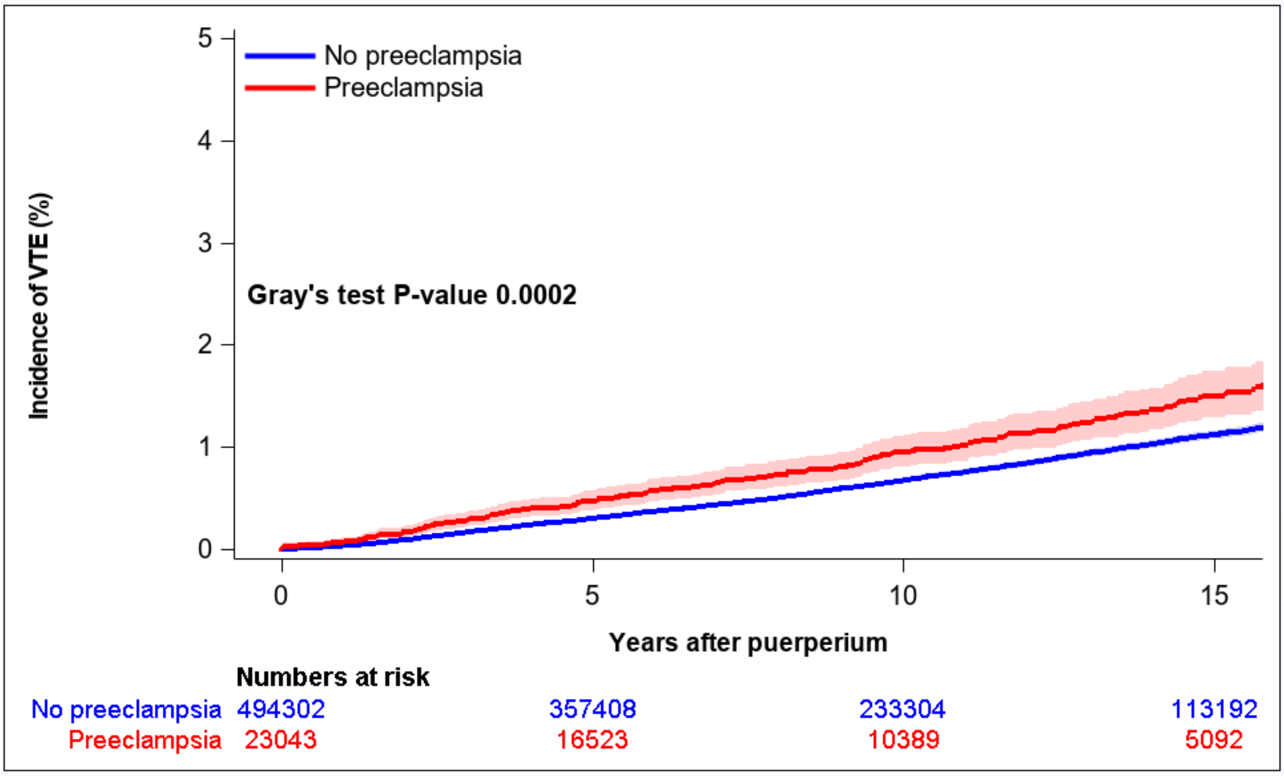

© 2023 Havers-Borgersen E et al. *JAMA Network Open.*

eFigure 2. Rates of Deep Vein Thrombosis, Pulmonary, and Overall Venous Thromboembolism Among Women With and Without Preeclampsia During Pregnancy, During the Puerperium, and Following the Puerperium. Adjusted for obesity, thrombophilia including antiphospholipid syndrome, heart failure, cancer, inflammatory and autoimmune diseases, and venous insufficiency.

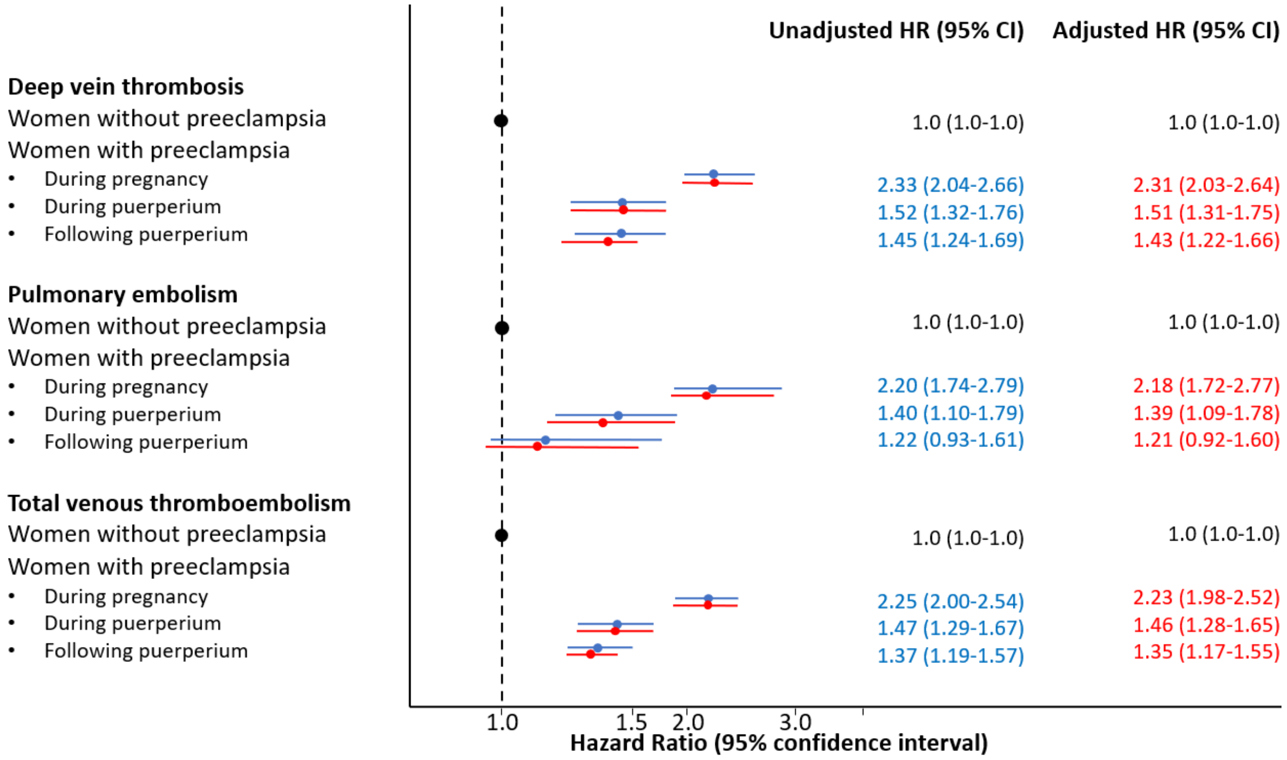

© 2023 Havers-Borgersen E et al. *JAMA Network Open.*

eFigure 3. Cumulative Incidences of Venous Thromboembolism Among Women Without Preeclampsia, With Early-Onset (<34 Weeks' Gestation) Preeclampsia and Late-Onset (≥34 Weeks' Gestation) Preeclampsia

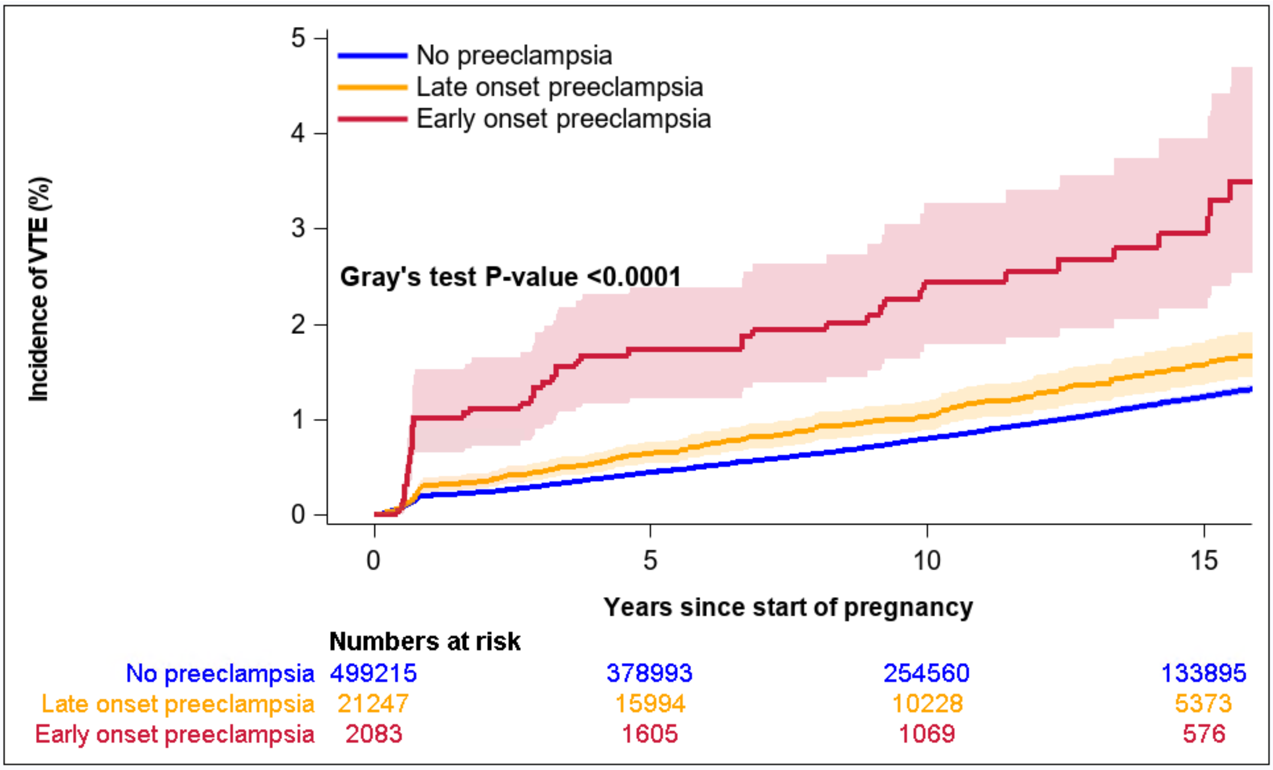

© 2023 Havers-Borgersen E et al. *JAMA Network Open.*

eFigure 4. Cumulative Incidences of Mortality Among Women With and Without Preeclampsia

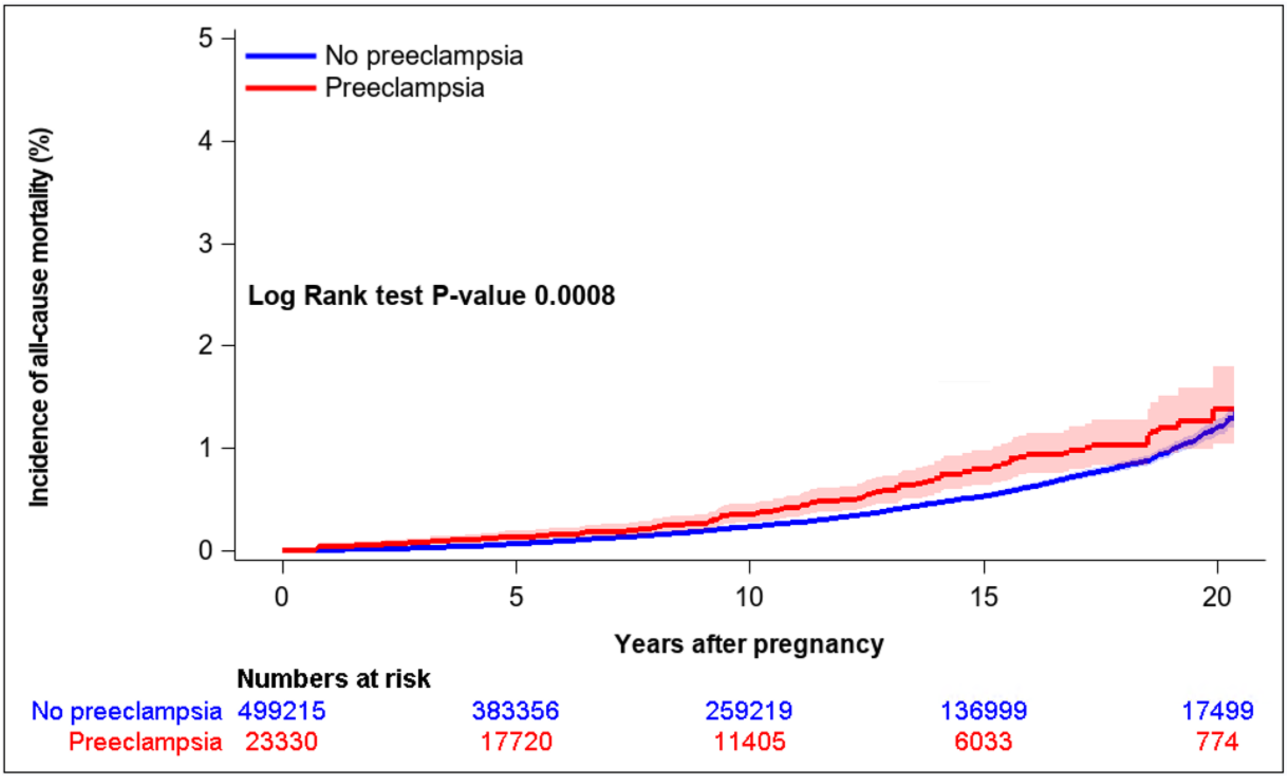

© 2023 Havers-Borgersen E et al. *JAMA Network Open.*
