# Supplementary material for: Preeclampsia and Long-Term Risk of Venous Thromboembolism
Source: JAMA Netw Open. 2023 Nov 17;6(11):e2343804. doi: 10.1001/jamanetworkopen.2023.43804 (PMC10656639; doi:10.1001/jamanetworkopen.2023.43804)
Supplement: Supplement 2. — Data Sharing Statement [file jamanetwopen-e2343804-s002.pdf]

Data Sharing Statement

Havers-Borgersen. Preeclampsia and Long-Term Risk of Venous Thromboembolism. *JAMA Netw Open*. Published November 20, 2023. doi:10.1001/jamanetworkopen.2023.43804

Data
Data available: No

Additional Information
Explanation for why data not available: Due to legislation on anonymity by Statistics Denmark, data is not publicly available, but it is possible to apply for access.
